# The controllable destabilization route for synthesis of low cytotoxic magnetic nanospheres with photonic response

**DOI:** 10.1038/s41598-017-11673-4

**Published:** 2017-09-12

**Authors:** Yulia I. Andreeva, Andrey S. Drozdov, Anna F. Fakhardo, Nikolay A. Cheplagin, Alexander A. Shtil, Vladimir V. Vinogradov

**Affiliations:** 1 ITMO University, Laboratory of Solution Chemistry of Advanced Materials and Technologies, Lomonosova St. 9, 191002 St. Petersburg, Russian Federation; 2 Department of Physical Electronics and Technology, St. Petersburg Electrotechnical University, Prof. Popova St. 38, St. Petersburg, 197376 Russian Federation; 3 Blokhin Cancer Center, Kashirskoye Shosse 24, Moscow, 115478 Russian Federation

## Abstract

We present a new approach for obtaining magnetic nanospheres with tunable size and high magnetization. The method is implemented via controllable destabilization of a stable magnetite hydrosol with glycerol, leading to the formation of aggregates followed by their stabilization with the citrate shell. This inexpensive, simple and easily scalable approach required no special equipment. The obtained samples were characterized by high stability and magnetization over 80 emu/g. Effects of synthetic conditions on physicochemical properties of nanospheres were monitored by hydrodynamic size, zeta potential, and polydispersity of magnetite aggregates. The size of the resulting aggregates varied between 650 nm and 40 nm, and the zeta potential from +30 mV to −43 mV by changing the ratio of the reagents. Under optimal conditions the clusters with a diameter of 80 nm were produced with a narrow size distribution ±3 nm. These characteristics allowed for optical response to the external magnetic field, thereby producing a magnetic photon liquid. Due to biocompatibility of the reagents used in the synthesis the nanospheres evoked a negligible cytotoxicity for human non-malignant and tumor cell lines. These results make new materials valuable in photonics and biomedicine.

## Introduction

Monodisperse magnetic nanospheres (MNS) are widely used in a variety of research and technological areas. Due to their unique physicochemical properties, the applications of these structures in biomedicine (e.g., for magnetic separation of bioobjects^1–4^, targeted drug delivery^5–10^, and MRI spectroscopy^11, 12^) is of particular interest. Also, MNS have prospects in optics and photonics^13^. Because of special requirements for these systems, studies were aimed at the synthesis of nanospheres with a hydrophilic functional surface to facilitate covalent cross-linking with biomolecules and stabilizers. The magnetic core consisting of aggregated nanoparticles provides high magnetization which is necessary for rapid manipulations and high signal sensitivity, the characteristics useful in photonic devices^14–17^ and biomedicine^18–20^. To date, two main approaches to obtain MNS have been pursued. First, a one-pot method implies the formation of nanoparticles and their aggregation during synthesis. In particular, the hydrothermal method for synthesis of nanospheres with a high magnetite content gives very narrow size distribution^21–23^. For instance, it is possible to obtain MNS stabilized by sodium citrate in an autoclave at high temperature (above 200 °C) and pressure (13 790 kPa)^24^. Using this approach 60–200 nm MNS have been generated. By changing the reaction conditions and the ratio of components and stabilizers, one can vary the textural and optical properties of resulting systems in a wide range. Although this approach could be used to produce the systems with high colloid stability and photonic properties, it requires special equipment, harsh conditions such as a high pressure and temperature, and carcinogenic chemicals such as ethylene glycol and diethylene glycol, thereby limiting possible application scenario. Another procedure relies on a polyol synthesis of nanospheres with a high degree of magnetization and a narrow dispersion^25^. This process includes the oxidation-reduction reaction between the metal precursor and liquid polyols, usually ethylene glycol, acting as polar solvents and reducing agents. In this procedure the hydrophilic magnetic nanocrystals are synthesized *in situ* and simultaneously self-organize into compact clusters, this, in turn, results in a high magnetic response of the clusters. Polyols strongly affect the size and morphology of particles, which greatly complicates the management of physicochemical properties of MNS. Although this approach produces the systems with a high colloid stability and photonic response, they require special equipment for synthesis. Also, carcinogens such as ethylene glycol and diethylene glycol are used, thereby limiting the biomedical applications of synthesized materials. The second approach presumes the use of previously prepared nanoparticles as building blocks for constructing larger aggregates^26, 27^. A sol-gel method^26^ implies that particles obtained at the first stage are covered with a silica coating, yielding nanospheres whose core consists of several magnetite particles with a silicon shell. This approach involves several stages and requires the second component that makes the synthesis much longer and contradicts the trend towards simplification of the composition. In addition, the silica shell significantly reduces the magnetic susceptibility of the material, making it hardly useful for magnetic delivery.All the above methods are considered ‘bottom-up’ approaches, in which the aggregation of ultrafine particles is achieved with stabilizers. In this study we present a new method for obtaining MNS by controllable destabilization of a stable magnetite hydrosol. This method leads to the formation of aggregates with various sizes followed by stabilization with the citrate shell. Our inexpensive, simple and easy-to-scale approach does not require any special equipment. Samples are highly stable. In addition to analyzing the sizes of the resulting structures, their stability and polydispersity, a particular attention is paid to 80 nm MNS that exhibit photonic properties at high concentrations in solution. At lower concentrations MNS behave similarly to magnetic nanoparticles (MNP) and quickly separate when an external magnetic field is applied. Rapid magnetic response and a negligibly low cytotoxicity provide evidence for a perspective of newly developed systems in photonics and biomedicine.

## Results and Discussion

### Preparation of MNS

**Figure 1:**
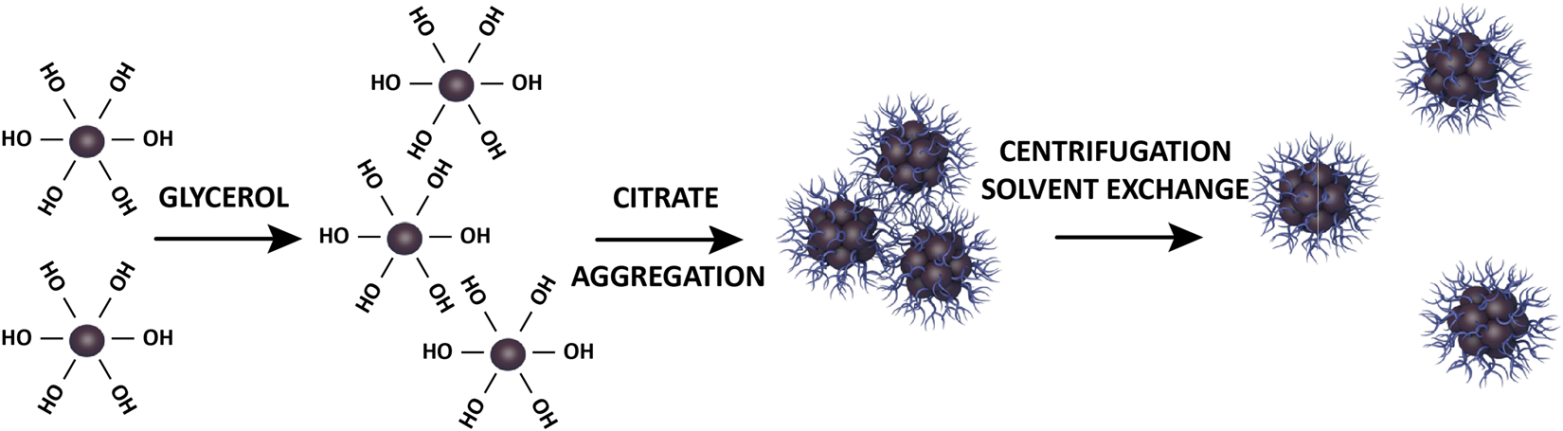
Controllable destabilization route for synthesis of MNS.

Synthesis of MNS was carried out using the newly developed route of controllable destabilization. The schematic diagram is shown in Fig. 1. The proposed strategy was to form magnetic nanoaggregates by manipulating with colloidal stability of the magnetite hydrosol, then to stabilize the formed aggregates by surface modification followed by removal of the destabilizing agent. As a result, a water-based stable colloidal MNS system is generated.

**Figure 2:**
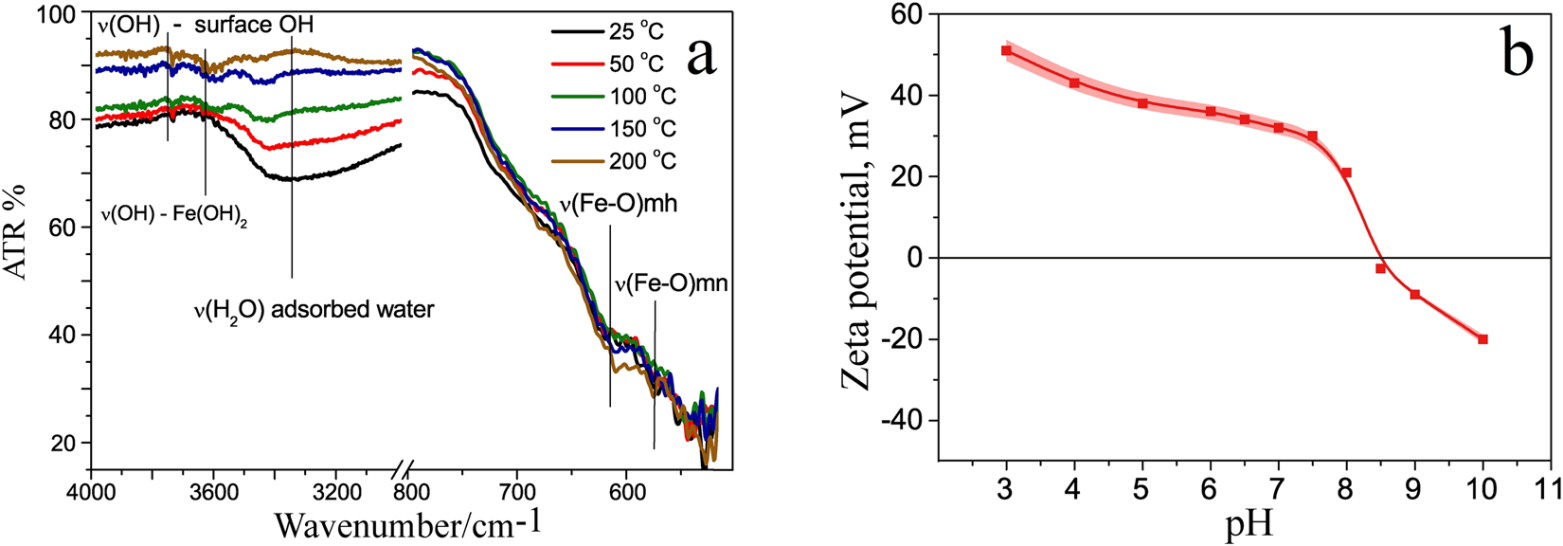
Characterization of MNP. Shown are IR-ATR spectra of MNP. Dehydration allows for distinguishing between different OH– vibration modes and vibration modes of adsorbed H_2_O molecules at the sample’s surface. Bands originating from magnetite (mn) and maghemite (mh) are given for comparison (**a**); Zeta potential and isoelectric point of the magnetite hydrosol (**b**).

To implement this method we used a stable magnetite hydrosol with uncoated nanoparticles described in detail^28^. The unmodified surface allows for sorbtion of different molecules and a wide variation of colloidal stability of nanoparticles by surface decoration. Furthermore, it is possible to achieve controllable destabilization in order to form bigger aggregates. The hydrosol consists of MNP 10 nm in diameter; size distribution is narrow. The surface of particles is highly hydrophilic due to OH- groups originating from magnetite and Fe(OH)_2_ on the particle’s surface (Fig. 2a). The presence of hydroxyl groups on the surface is sufficient to shift the isoelectric point of the material to basic pH values (Fig. 2b) and achieve the value of zeta potential +36 mV at pH = 7 resulting in excellent colloidal stability.

The magnetite hydrosol was mixed with glycerol to destabilize the colloidal system. This cheap, non-toxic, polybasic alcohol completely miscible with water possesses high viscosity (1200 Pa * sec) and a twofold lower relative permittivity (39.1 and 80 for glycerol and water, respectively). The destabilizing effect of glycerol is based on its ability to form hydrogen bonds. Polyols are known to adsorb on the surface of magnetite nanoparticles in a competitive way by forming the hydrogen bonds with surface hydroxyl groups^29^. Since the energy of glycerol adsorption on the magnetite surface is higher than that of water (the values of energy of binding are 0.7 eV for water and 1 eV for glycerol per functional group^29^), glycerol added to the magnetite hydrosol adsorbs on the magnetite surface. Consequently, the zeta potential of the surface decreases. Due to a lower relative permittivity, glycerol further diminishes the value of the zeta potential of the system (see ESI Fig. 1S). Importantly, the addition of glycerol to the magnetite hydrosol does not lead to coagulation of nanoparticles due to an increase of viscosity of the system (ESI Fig. 2S). Glycerol is completely mixable with water, therefore its ability to solvate highly polar ionic compounds such as inorganic salts is limited by a low dipole moment (0.28 Db at 20 °C). The biocompatible sodium citrate was chosen as a coating for stabilization of aggregates. This salt of triprotic acid is known to coordinate the MNP surface via the hydroxyl group. When coordinated to magnetite, the citrate residue orients its carboxyl groups into solution providing a negative charge for the particle’s surface^24^. The addition of the citrate solution to the magnetite sol in a water-glycerol mixture promotes aggregation of MNP. Coordination of citrate residues on the magnetite surface increases Na^+^ concentration near the surface of nanoparticles, thereby lowering the ability of the water-glycerol mixture to solvate the nanoparticles and promoting the growth of aggregates. Finally, the mixture is centrifuged and washed with ethanol to remove glycerol. Exchange of the solvent for water leads to electrostatic repulsions of aggregates and formation of stable MNS hydrosols.

**Figure 3:**
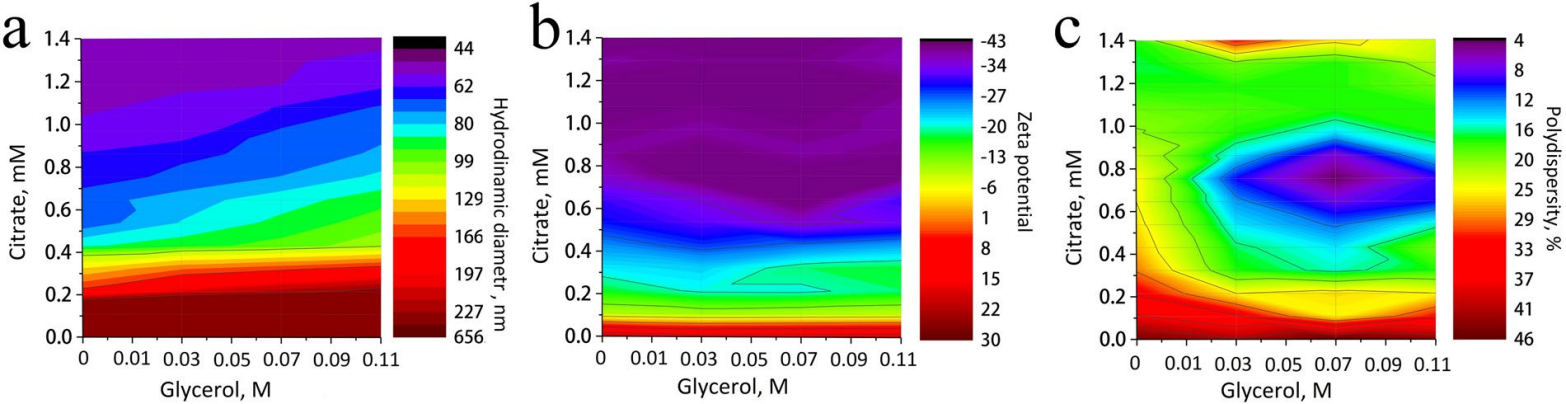
Changes of the hydrodynamic diameter (**a**), zeta potential (**b**) and polydispersity of MNS (**c**) depending on the concentration of glycerol and citrate in the system. The concentration of MNS was 15 mg/mL.

Next, we addressed the questions: how does the size of aggregates depend on the conditions of controllable system destabilization? What conditions are optimal for stability? What are the requirements for the ratios of components to achieve minimum size distribution? In so doing we investigated the effects of conditions of controllable MNP destabilization on the size, charge, and size distribution of MNS by dynamic light scattering (DLS) technique. Average hydrodynamic diameter, zeta potential, and polydispersity were measured at various glycerol and citrate concentrations (the concentration of particles was constant, that is, 15 mg/mL). The addition of glycerol to the magnetite hydrosol without citrate capping followed by centrifugation and washing with ethanol led to the formation of big aggregates with average diameter 640 nm due to coagulation of nanoparticles accompanied by a decreased zeta potential (Fig. 3a,b). The charge of MNP coated only by OH-groups was insufficient to maintain the colloidal stability of the system and to redisperse MNP in aqueous media after removal of ethanol. Therefore, sedimentation of these aggregates was quick; no stable suspension was formed. The addition of citrate ions to MNP in the water-glycerol media led to their coordination on the surface of aggregated clusters and a shift of the surface charge from positive to negative (Fig. 3b). At the same time, with the increase of the value of zeta potential a reduction of the hydrodynamic diameter of aggregates was observed (Fig. 3a). The size of the produced aggregates can be tuned in the range 650–40 nm, and the value of zeta potential from +30 mV to −43 mV, by changing the ratio of reagents. The reduction of the aggregates’ size with elevation of citrate concentration indicates that adsorption of citrate ions on the surface of aggregates stabilizes the aggregates formed during destabilization. At bigger citrate concentration the nanoaggregates are disrupted into smaller parts, down to individual MNP. Further increase of the amounts of citrate and glycerol above 0.7 mM does not affect the particles’ charge. This fact evidences the saturation of the double electric layer with the citrate ions. Apart from the hydrodynamic parameters and the charge of particles, another important factor is the polydispersity of the nanoaggregate size determined by the ratio of the measured peak width to its height. Figure 3c shows that the systems with minimum dispersion were formed within the concentration range corresponding to 0.05–0.09 M glycerol and 0.6–0.9 mM citrate. At these concentrations the nanoclusters with the hydrodynamic diameter 80 ± 3 nm were formed (Fig. 3c). One reason for this behavior is the fact that, within these concentration ranges, we observed maximum values of zeta potential. On the other hand, the size of the clusters was in the zone of optimal values; above or below this zone we registered either an abrupt coagulation of the system and formation of big aggregates, or destruction of clusters due to an excess of citrate. In both situations the system broke apart into individual particles with a broadened particle’s size distribution.

### Textural, magnetic and photonic properties of MNS

**Figure 4:**
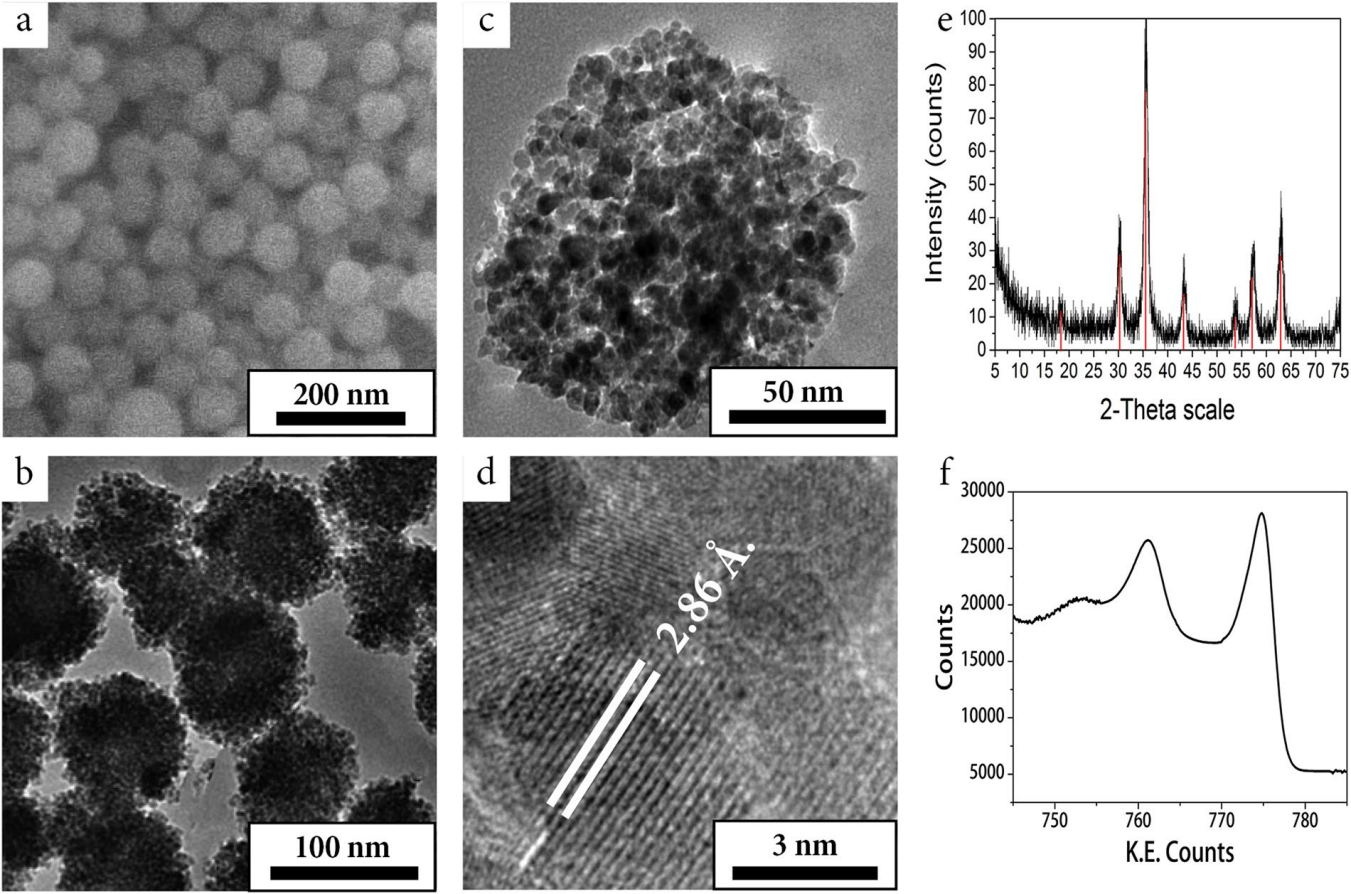
Characteristics of MNS. SEM (**a**), TEM (**b**,**c**) and HRTEM (**d**) images of 80 nm MNS. XRD (**e**) and XPS (**f**) analyses show the presence of magnetite crystalline phase.

SEM of the nanoclusters produced at optimal conditions demonstrated that the formed aggregates had nearly spherical shape with uniform size distribution (Fig. 4a). TEM analysis of the aggregates (Fig. 4b,c) is in good agreement with SEM data demonstrating that the aggregates were loose clusters of magnetite nanoparticles (Fig. 4c), detectable with a high-resolution TEM (HRTEM) (Fig. 4d). The aggregates represented 10 nm magnetite nanocrystals with an interplanar distance 2.86 *Å* typical for magnetite^30^. The X-ray diffraction (Fig. 4e) also proved the presence of magnetite with average crystallite size 10 nm. The X-ray photoelectron spectrum (Fig. 4f) showed peaks at 711.8 eV and 724.8 eV that are in agreement with known values of respective Fe2p3/2 and Fe2p1/2 oxidation states of Fe in Fe_3_O_4_. Obviously, the resulting materials have a high surface area and can be used for adsorption of guest molecules, e.g., drugs.

**Figure 5:**
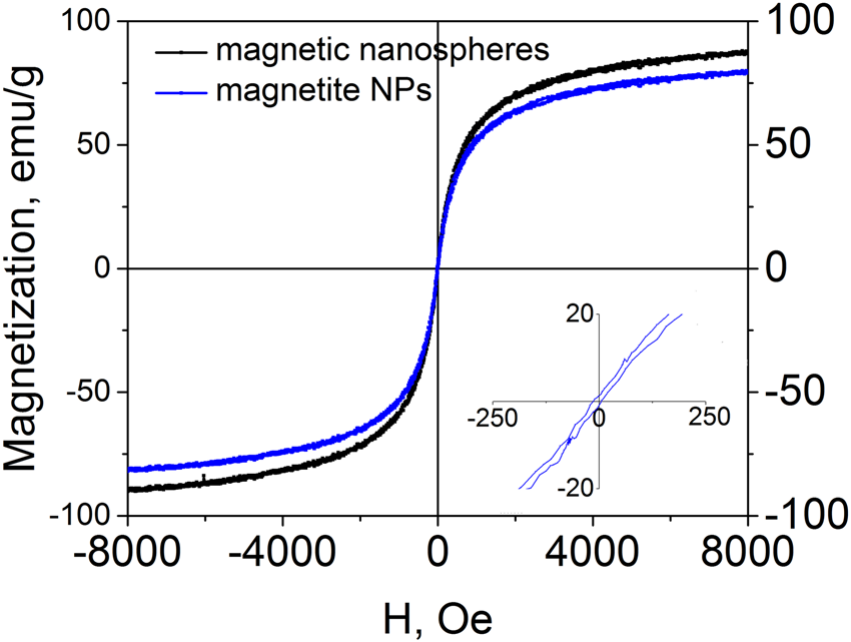
Magnetization curves of the magnetite hydrosol and 80 nm MNS at room temperature.

The magnetic curves of synthesized 80 nm MNS showed almost complete absence of hysteresis (Fig. 5), demonstrating a superparamagnetic behavior at room temperature. The specific magnetization of MNS is 80 emu/g. Such a high value can be explained by low content of organic components in nanoaggregates and a high amount of magnetite.

**Figure 6:**
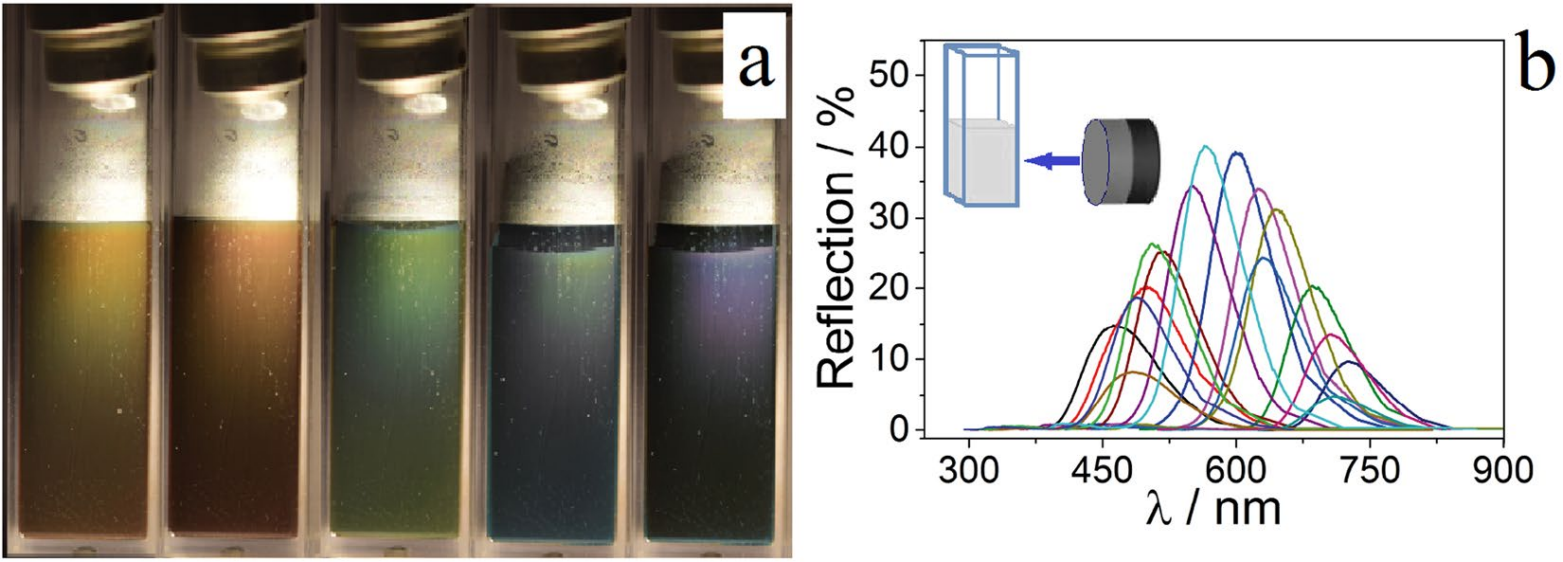
Photonic properties. Change of color of the magnetic photonic liquid along with the decrease (from 3 cm to 1 cm) of the distance between the magnet and the sample (**a**). Reflection spectra of the magnetic colloid. The angle of incidence is 8° (**b**).

Due to the high magnetization value, excellent colloidal stability and low size polydispersity the colloid solution of synthesized MNS demonstrated the properties of magnetic photon liquids, i.e., change of the reflectance spectrum under the applied magnetic field. Figure 6a displays visual representation of samples subjected to the different external magnetic field strength by alternating the distance between the neodymium magnet and the cuvette. The color of the sample changed from red to violet when the magnetic field is increased from 200 G to 680 G (the magnet was moved closer to the sample from 3 cm to 1 cm). The spectrum underwent a characteristic blue-shift of the reflection maximum from 720 nm to 445 nm as the magnet approached (Fig. 6b). The mechanism of this phenomena is described in the next section.

### Magnetophotonic phenomena in the colloid: a background

The newly synthetized magnetic nanoaggregates can be regarded as superparamagnetic nanocrystal clusters. Each particle is comprised of tiny magnetite crystallites with high magnetization value and superparamagnetic behavior. In the absence of magnetic field the magnetic moments of the cluster constituents are disordered, so that the clusters have zero net magnetic moment (Fig. 5). The value of zeta potential ensures electrostatic repulsion of the clusters maintaining the high colloidal stability of the system. When the external magnetic field is applied the magnetization of crystallite constituents of the colloid particle lines up along the field direction. Hence, each cluster acquires the induced magnetic moment *μ* that depends on the field strength and particle’s dimensions. Consequently, particles can be treated as the magnetic dipoles. Consider the two types of interactions between the dipoles and the external magnetic field, namely the dipole-field and dipole-dipole interactions. As an outcome of the dipole-field interaction, the dipoles line up along the field direction. If there is also a field gradient applied to the colloid, clusters fall under the packing force that drives them towards field maximum. After a certain time the packing force induces phase separation of the colloid. The dipole-dipole interaction magnitude is described by the equation:

$$F=\frac{3{|\mu |}^{2}(1-3\,{\cos }^{2}\,\vartheta )}{{d}^{4}}$$

where is *θ* the angle between the direction of the magnetic moment of the particle and the line connecting the centers of two particles; *d* stands for the distance between two particles. Depending on *θ*, the dipole-dipole interaction can be either attractive or repulsive. Two adjacent particles oriented along the magnetic field line are subjected to the attraction force:

$$F=\frac{6{|\mu |}^{2}}{{d}^{4}}$$

whereas two neighboring particles undergo the repulsion force:

$$F=\frac{3{|\mu |}^{2}}{{d}^{4}}$$

**Figure 7:**
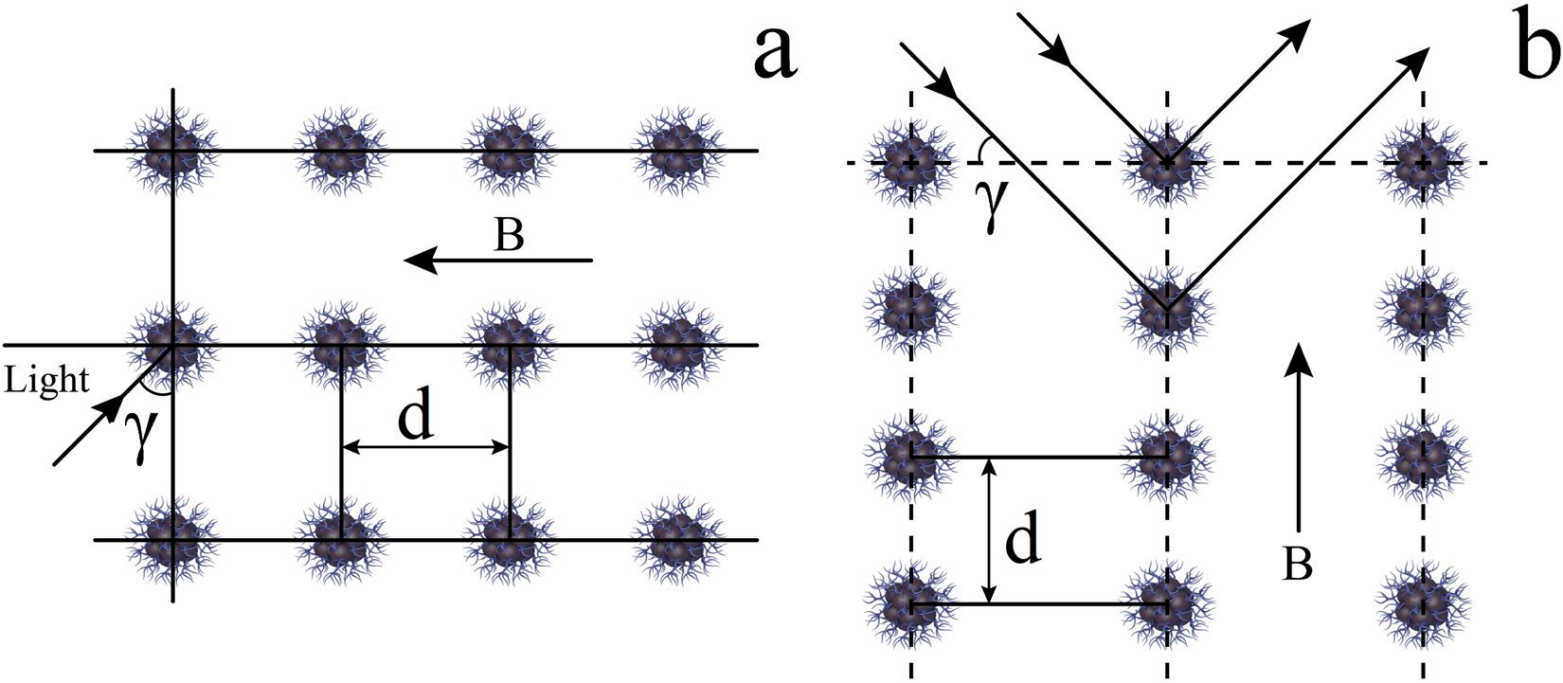
Arrangement of particles into chains. Section of the chain structure. The external magnetic field affects MNS by attraction and repulsion forces (**a**). Schematic interaction of light with the chain structure. Due to high ordering of MNS lattice the Bragg’s law is applicable (**b**). *d* is the distance between nanoparticles, *B* is a magnetic field.

Such a twofold coupling with critical angle 8° results in arrangement of particles into chains (Fig. 7). Citrate shells also contribute to the effect of particle repulsion. The electrostatic force Fe increases the degree of repulsion between MNS.

Consequently, force equilibrium is established in the colloid. This equilibrium is governed by the magnitude of the external magnetic field. Since the colloid particles have almost equal dimensions, and because of uniformity of the external magnetic field, the forces between particles are generally of the same magnitude. This means that the chains in which the particles are organized can be regarded as periodic. Along such structures the Rayleigh-Bloch waves can propagate^15^. Hence, this structure possesses a rejection band with the center frequency that can be roughly estimated using Bragg’s condition:

$$d\,{\sin }\,\gamma =\lambda $$

where *γ* is the angle of incidence of the wave upon the array of chain structures, *d* is an integer, and *λ* is wavelength of light (Fig. 7). With certain particle size as well as chain periodicity the rejection band can manifest itself in the optical reflection spectrum. Variation of the magnetic field strength changes the periodicity of the chain structure due to new force equilibriums. This entails a shift of the center wavelength of the rejection band. Relative optical spectra of the colloid are shown in Fig. 6b.

### Separating nanospheres from the solution

**Figure 8:**
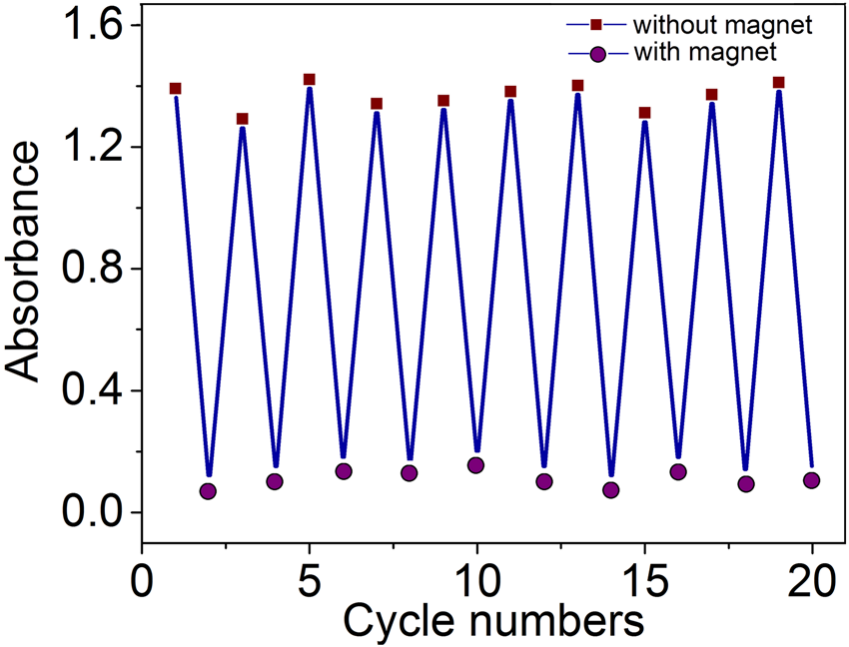
Absorbance of 0.15 mg/mL MNS at 355 nm after intermitting applications of the magnetic field. Nanoparticles were quickly attracted to the magnet making the solution transparent but became dispersed in the absence of the field.

When we dissolved MNS by a factor of 100 (down to 0.15 mg/mL), the degree of particle magnetization remained unchanged. Upon interaction with the magnetic field the solution behaved differently. If the colloid was completely attracted to the magnet, the photonic properties were lost. Therefore, the solution was no longer a magnetic fluid but a system in which magnetic particles were dispersed in water. However, the particles fully attracted to the magnet can be reversibly dispersed in water by gentle shaking. To confirm the reversibility of this process, UV-Vis spectra were recorded. Figure 8 shows good regeneration of the system in the course of cyclic measurements of the absorption spectrum of the solution.

### Stability of the synthesized nanospheres

We evaluated the applicability of newly synthesized materials for practical use. Citrate ions are known to form stable chelate complexes with metal ions that can lead to leaching of iron ions from the magnetite core. Incubation of the colloid system at pH 3–12 in anaerobic conditions caused no leach of iron ions over 24 h; at pH 2 and 1 the leach of iron reached 0.07% and 0.16% wt., respectively. In contrast, MNS demonstrated a low colloidal stability at pH  < 4.5, (see ESI Figs 3S and 4S). Evaluation of the colloidal stability as a function of pH demonstrates, that at pH ≤ 4.5 the nanoagregates undergo coagulation due to a low value of its zeta potential. For higher pH values excellent colloidal stability was observed with no signs of coagulation even after one week.

### Cytotoxicity of MNS

**Figure 9:**
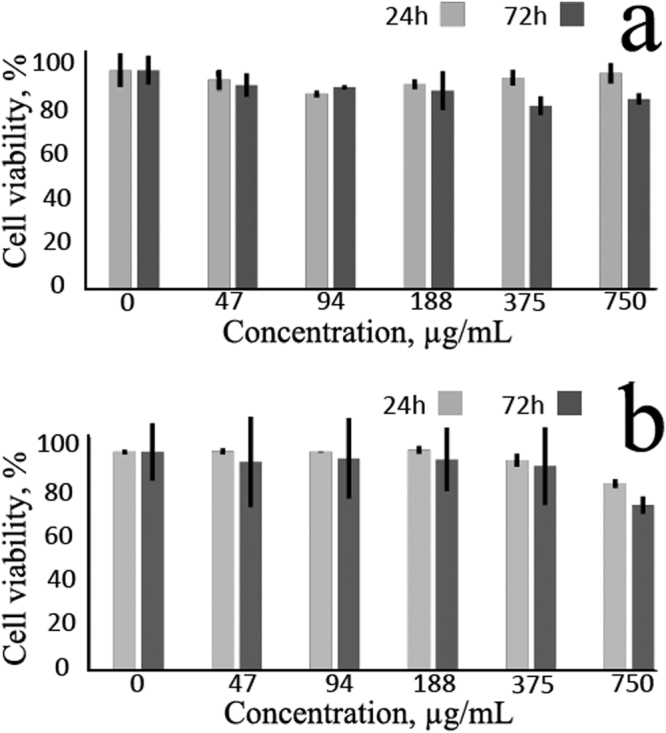
Weak antiproliferative effect of MNS on (**a**) HELF and (**b**) HeLa cells after 24 h and 72 h exposure. Shown are average values of 3 measurements with standard deviations.

It is worth noting that only the reagents approved for parenteral administration in humans were used in the synthesis. Nevertheless, we tested the cytotoxicity of new 80 nm MNS. Figure 9 shows that, at 47–750 *μ*g/mL, MNS evoked little-to-no cytotoxic effect on HELF or HeLa cells after a 72 h continuous exposure (time sufficient for 3 doublings of the culture). By 72 h the biggest decrease of MTT conversion, a marker of cell growth retardation, was only 13% for HELF cells and 24% for HeLa cells, respectively. No morphological signs of cell death were observed (not shown) indicating that even at maximum tested concentrations MNS were tolerable. By 24 h cell growth retardation was even less pronounced. Importantly, our materials were less toxic to human cells than previously reported compounds^31^ probably due to the use of glycerol instead of the toxic ethylene glycol. Indeed, at 188 *μ*g/mL MNS (the concentration similar to the one used in ref. 31 the survival rates of HELF and HeLa cells were 91% and 97%, respectively. These values exceed the results in ref. 31; moreover, in our experiments the time of cell exposure was as long as 72 h compared to only 12 h, strongly supporting the idea that our synthetic route yields MNS with superior biocompatibility.

## Conclusions

We aimed at a strategy of a quick, inexpensive, environmentally safe and reproducible synthesis of biocompatible MNS with broad research and technological potential. The new approach is based on controllable destabilization of the magnetite hydrosol. The particle size can be tuned in a wide range of 40–650 nm by varying the concentrations of glycerol (a destabilizing agent) and citrate (stabilizes the forming aggregates). Systems with minimum size distribution were generated with 0.05–0.09 M glycerol and 0.6–0.9 mM citrate. Within these ratios, 80 ± 3 nm clusters were obtained. MNS are composed of numerous primary 10 nm magnetite nanocrystals possessing superparamagnetic properties and a high (80 emu/g) magnetization. Water colloid solutions of 15 mg/mL MNS behave as a magnetic photon liquid that changes its reflectance spectra upon application of the external magnet. At lower concentrations (0.15 mg/mL) MNS can be easily separated with the magnet which might be useful in the design of magnetic drugs. Importantly, due to the citrate groups attached to the surface, the magnetite particles have excellent water dispersibility and dispersing stability. Furthermore, only the reagents approved for administration in humans were used in the synthesis. Subsequently, our systems were compatible with cultured human cells even at high concentrations after a prolonged treatment, making these materials valuable for various applications, in particular, photonics and biomedicine. Finally, the developed method can be useful in generating the clusters of other metal oxides with desired physicochemical properties.

## Experimental Details

### Materials

Iron (II) chloride tetrahydrate, iron (III) chloride hexahydrate, ammonia aqueous solution, glycerol, sodium citrate pentahydrate, ethanol 99%, and MTT powder were obtained from Sigma-Aldrich. Dimethyl sulfoxide (DMSO) was from VWR. Phosphate buffered saline (PBS) tablets were purchased from Gibco.

### Synthesis of MNS

To obtain MNS, a stable magnetite hydrosol was used. Pure ferria hydrosol was prepared ultrasonically from iron (II) chloride tetrahydrate and iron (III) chloride hexahydrate as described^28^. The mass fraction of nanoparticles with average particle size 10 nm in the resulting sol was 2 wt%. To initiate the process of controllable destabilization of the system, 0.27 mM of freshly prepared mag-netite hydrosol was treated with different amounts of glycerol (up to 0.11 M) and stirred for 5 min (500 rpm) at 25 °C. Different amounts of sodium citrate dissolved in 2 mL water were added (up to 1.4 mM) to prevent further aggregation and stabilization of the formed clusters. The resulting mixtures were stirred for 15 min (500 rpm) at 25 °C. To wash the nanospheres from glycerol, the solution was treated with 2 mL ethanol and centrifuged (11.000 rpm, 5 min). The precipitate was washed with a mixture of alcohol and water (3:1) 5 times and dispersed in 5 mL water to yield MNS concentration 0.07 mg/mL. The resulting hydrocolloid of MNS was evaporated to reach 15 mg/mL. The systems with photonic response were generated at 0.05–0.09 M glycerol and 0.6–0.9 mM citrate. These ranges were critical because lower or bigger concentrations of these reagents led to a decrease or disappearance of photonic properties (see below).

### Iron ions leach determination

In order to evaluate the stability of magnetite nanospheres against iron ions leaching 2 mL of the colloid solution were mixed placed into plastic test tube and the pH was adjusted to the required pH with 1 M HNO_3_ or KOH, purged with purified N_2_, sealed and shaked at room temperature for 24 h. The colloid solution was withdrawn with a syringe and immediately filtered through a membrane of 220 nm pore size. Dissolved Fe was determined by using the o-phenanthroline method at a wavelength of 508 nm^32^.

### MTT Assay

To evaluate the cytotoxicity of 80 nm MNS the human non-malignant embryonic lung fibroblasts (HELF) and tumor HeLa cell lines (Biolot, St.Petersburg, Russia) were maintained in Eagle’s medium (Biolot) supplemented with 10% fetal bovine serum (Gibco) and 50 *μ*g/mL gentamycin (Biolot) at 37 °C, 5% CO_2_. Cells at logarithmic phase of growth were plated (5–10 * 10^3^/well) into 96-well plates overnight and then treated for 24 h or 72 h with MNS resuspended directly in the culture medium at final concentrations 47–750 *μ*g/mL. The volume of added MNS from the stock suspension in PBS was <5% of total volume of the culture medium in the wells. After the completion of cell exposure the medium with particles was discarded, and 200 *μ*l MTT (3-(4,5-dimethylthiazol-2-yl)-2,5-diphenyltetrazolium bromide; 0.5 mg/mL) solution in PBS was added to each well for 1.5 h. Then MTT solution was aspirated, and formazan granules were dissolved in 200 *μ*l DMSO. Optical density was measured at 570 nm on a Tecan Infinite 50 spectrophotometer. Cell viability was calculated as the percentage of optical densities in wells with each concentration of MNS normalized to the optical density of untreated cells (100%).

### Characterization

IR-ATR spectra were recorded on an FTIR Perkin–Elmer System 2000 spectrometer utilizing Heated Golden Gate ATR accessory (product of SPECAC) with a diamond ATR crystal, using single reflectance and ZnSe focus lenses. Sixty four scans for the background (air) and the samples were recorded. The sample was heated from room temperature to 50, 100, 150 and 200 °C *in situ*; the spectrum was recorded 15 min after stabilization of the acquired temperature. The hydrodynamic diameter and zeta potential were measured with DLS using Photocor Compact Z. Polydispersity was calculated as the relation of the peak width to its maximal amplitude position, measured by DLS. Samples for transmission electron microscopy (TEM) were obtained by dispersing a small probe in ethanol to form a homogeneous suspension. Then, a suspension drop was placed onto a copper mesh covered with carbon for TEM analysis (FEI TECNAI G2 F20, operating voltage 200 kV). For scanning electron microscopy (SEM) the resulting samples were dried in vacuo for 1 h and examined with Tescan VEGA 3. The crystalline phase and crystallinity of samples were measured by X-ray diffraction (Bruker D8 Advance) using Cu Ka radiation (*λ* = 1.54 *Å*); the samples were scanned for 2 h at a rate of 0.5 degrees per minute. MRI reflectance spectra were recorded on a Shimadzu UV-2600 spectrometer supplied with ISR-2600Plus integrating sphere (reference wavelength 355 nm, reflection angle 0°). Absorption was measured on an Agilent Technologies Cary 8454 UV-Vis spectrophotometer. XPS spectra were recorded on an ESCALAB Xi X-ray Photoelectron Spectrometer.

## Electronic supplementary material

Supplementary information

## References

[1] Braiek M (2016). Boron-doped diamond electrodes modified with fe_3_o_4_@Au magnetic nanocomposites as sensitive platform for detection of a cancer biomarker, interleukin-8. Electroanal..

[2] Hejazian M, Li W, Nguyen N-T (2015). Lab on a chip for continuous-flow magnetic cell separation. Lab on a Chip.

[3] He J, Huang M, Wang D, Zhang Z, Li G (2014). Magnetic separation techniques in sample preparation for biological analysis: a review. J. pharmaceutical biomedical analysis.

[4] Quaresma P (2014). Star-shaped magnetite@ gold nanoparticles for protein magnetic separation and sers detection. RSC Adv..

[5] Mou X, Ali Z, Li S, He N (2015). Applications of magnetic nanoparticles in targeted drug delivery system. J. nanoscience nanotechnology.

[6] Shabanova EM, Drozdov AS, Ivanovski V, Suvorova II, Vinogradov VV (2016). Collagenase@magnetite: proteolytic composite for magnetically targeted minimally invasive surgery. RSC Adv..

[7] Mody VV (2014). Magnetic nanoparticle drug delivery systems for targeting tumor. Appl. Nanosci..

[8] Hola K, Markova Z, Zoppellaro G, Tucek J, Zboril R (2015). Tailored functionalization of iron oxide nanoparticles for mri, drug delivery, magnetic separation and immobilization of biosubstances. Biotechnol. advances.

[9] Ulbrich K (2016). Targeted drug delivery with polymers and magnetic nanoparticles: covalent and noncovalent approaches, release control, and clinical studies. Chem. reviews.

[10] Drozdov, A. S., Vinogradov, V. V., Dudanov, I. P. & Vinogradov, V. V. Leach-proof magnetic thrombolytic nanoparticles and coatings of enhanced activity. *Sci*. *reports* **6**, 28119 (2016). 10.1038/srep28119 PMC4913305 27321930

[11] Thomas R, Park I-K, Jeong YY (2013). Magnetic iron oxide nanoparticles for multimodal imaging and therapy of cancer. Int. journal molecular sciences.

[12] Kinsella, J. M. *et al*. Enhanced magnetic resonance contrast of Fe_3_O_4_ nanoparticles trapped in a porous silicon nanoparticle host. *Adv*. *Mater*. **23** (2011). 10.1002/adma.201101877 PMC3548421 21842475

[13] Bhatt H (2016). Magnetic field dependent resonant light scattering by magnetic spheres in a magnetizable medium. Nanosyst. Physics, Chem. Math..

[14] Li F, Josephson DP, Stein A (2011). Colloidal assembly: the road from particles to colloidal molecules and crystals. Angewandte Chemie Int. Ed..

[15] Hu H, Chen C, Chen Q (2013). Magnetically controllable colloidal photonic crystals: unique features and intriguing applications. J. Mater. Chem. C.

[16] Zhao Y, Shang L, Cheng Y, Gu Z (2014). Spherical colloidal photonic crystals. Accounts chemical research.

[17] Pu S, Dong S, Huang J (2014). Tunable slow light based on magnetic-fluid-infiltrated photonic crystal waveguides. J. Opt..

[18] Wen C-Y (2013). Quick-response magnetic nanospheres for rapid, efficient capture and sensitive detection of circulating tumor cells. ACS nano.

[19] Farkas K, Földesi I, Illés E, Tombácz E, Tóth I (2015). Examination of hemocompatibility of magnetite nanoparticles designed for biomedical use. Clin. Chem. Lab. Medicine.

[20] Chen Y-W (2016). Magnetite nanoparticle interactions with insulin amyloid fibrils. Nanotechnol..

[21] Zhuang, L. *et al*. Hydrophilic magnetochromatic nanoparticles with controllable sizes and super-high magnetization for visualization of magnetic field intensity. *Sci*. *reports* **5**, 17063 (2015). 10.1038/srep17063 PMC4655409 26593643

[22] Jiang, L. *et al*. Preparation and characterization of poly (glycidyl methacrylate)-grafted magnetic nanoparticles: Effects of the precursor concentration on polyol synthesis of Fe_3_O_4_ and [PMDETA]_0_/[CuBr_2_]_0_ ratios on SI-AGET ATRP. *Appl. Surf. Sci.* **357**, 1619–1624 (2015).

[23] Abbas, M. *et al*. Highly stable-silica encapsulating magnetite nanoparticles (Fe_3_O_4_/SiO_2_) synthesized using single surfactantless-polyol process. *Ceram. Int.* **40**, 1379–1385 (2014).

[24] Wang W, Tang B, Ju B, Zhang S (2015). Size-controlled synthesis of water-dispersible superparamagnetic fe_3_o_4_ nanoclusters and their magnetic responsiveness. RSC Adv..

[25] Fu J (2016). Formation of colloidal nanocrystal clusters of iron oxide by controlled ligand stripping. Chem. Commun..

[26] Wang S, Tang J, Zhao H, Wan J, Chen K (2014). Synthesis of magnetite–silica core–shell nanoparticles via direct silicon oxidation. J. colloid interface science.

[27] Ojha K, Anjaneyulu O, Ganguli AK (2014). Graphene-based hybrid materials: synthetic approaches and properties. Curr. Sci..

[28] Drozdov AS, Ivanovski V, Avnir D, Vinogradov VV (2016). A universal magnetic ferrofluid: Nanomagnetite stable hydrosol with no added dispersants and at neutral ph. J. colloid interface science.

[29] Aschauer U, Selloni A (2015). Adsorption of biomedical coating molecules, amino acids, and short peptides on magnetite (110). The J. chemical physics.

[30] El Ghandoor H, Zidan H, Khalil MM, Ismail M (2012). Synthesis and some physical properties of magnetite (fe_3_o_4_) nanoparticles. Int. J. Electrochem. Sci.

[31] Liu J (2009). Highly water-dispersible biocompatible magnetite particles with low cytotoxicity stabilized by citrate groups. Angewandte Chemie.

[32] Fortune W, Mellon M (1938). Determination of iron with o-phenanthroline: a spectrophotometric study. Ind. & Eng. Chem. Anal. Ed..

