# Supplementary material for: The controllable destabilization route for synthesis of low cytotoxic magnetic nanospheres with photonic response
Source: Sci Rep. 2017 Sep 12;7:11343. doi: 10.1038/s41598-017-11673-4 (PMC5595919; doi:10.1038/s41598-017-11673-4)
Supplement: Supplementary file 1 — Supplementary information [file 41598_2017_11673_MOESM1_ESM.pdf]

Supplementary Information

The controllable destabilization route for synthesis of low cytotoxic magnetic nanospheres with photonic response

Yulia I. Andreeva[1], Andrey S. Drozdov[1]*, Anna F. Fakhardo[1], Nikolay A. Cheplagin[2], Alexander A. Shtil[3], and Vladimir V. Vinogradov[1]*

[1]ITMO University, Laboratory of Solution Chemistry of Advanced Materials and Technologies, 9 Lomonosov Street, St. Petersburg 191002, Russian Federation;

[2]Department of Physical Electronics and Technology, St. Petersburg Electrotechnical University, 38 Prof. Popov Street, St. Petersburg 197376, Russian Federation;

[3]Blokhin Cancer Center, 24 Kashirskoye Shosse, Moscow 15478,
Russian Federation.

*drozdov@scamt.ru, vinogradov@scamt.ru.

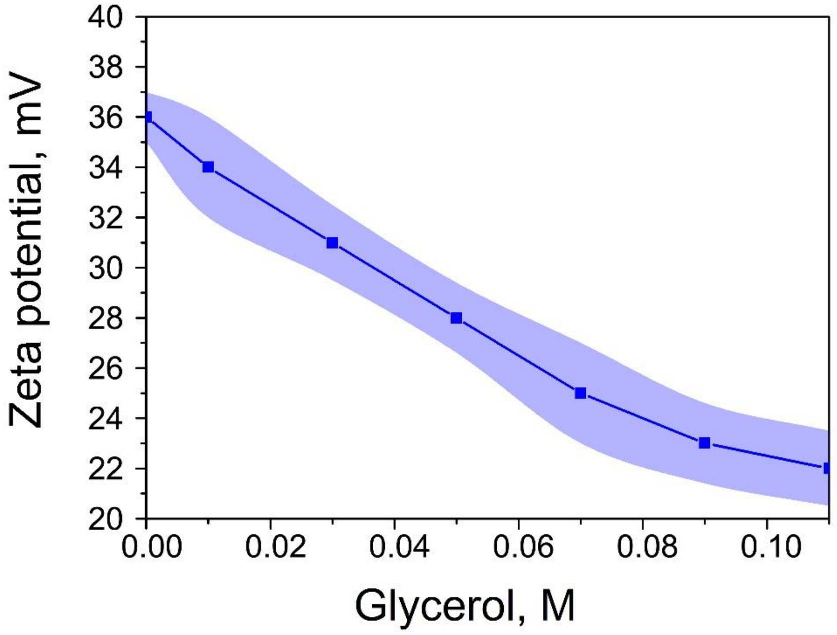

Figure 1S. Dependence of zeta potential on glycerol concentration. Increase of glycerol concentration leads to decrease of zeta potential value.

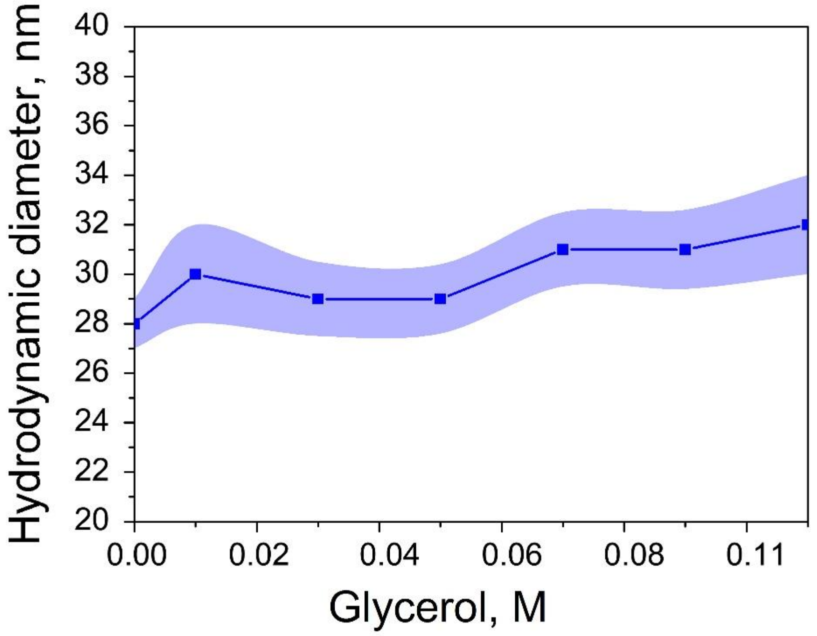

Figure 2S. Dependence of the hydrodynamic diameter on glycerol concentration.

The hydrodynamic diameter of magnetite hydrosol does not depend on glycerol concentration.

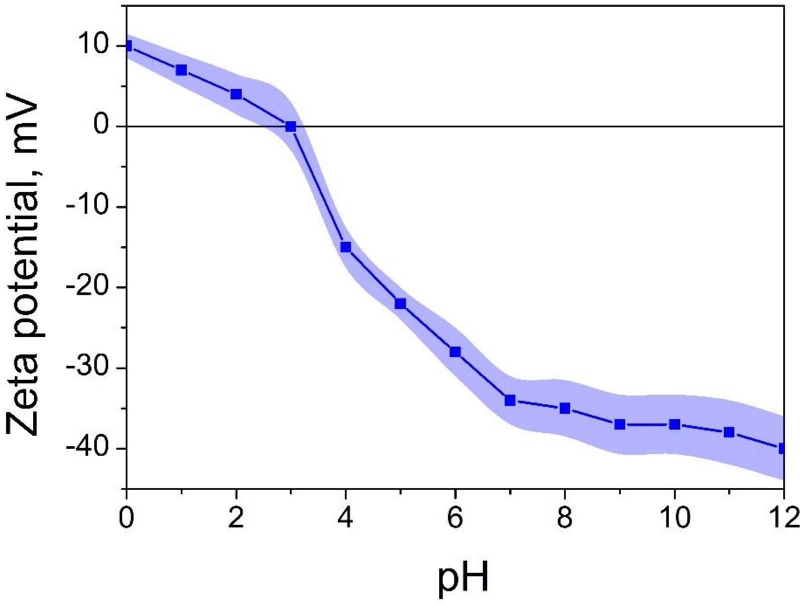

Figure 3S. Zeta potential of photon crystals as a function of pH.

The isoelectric point of the crystals is at pH 3.

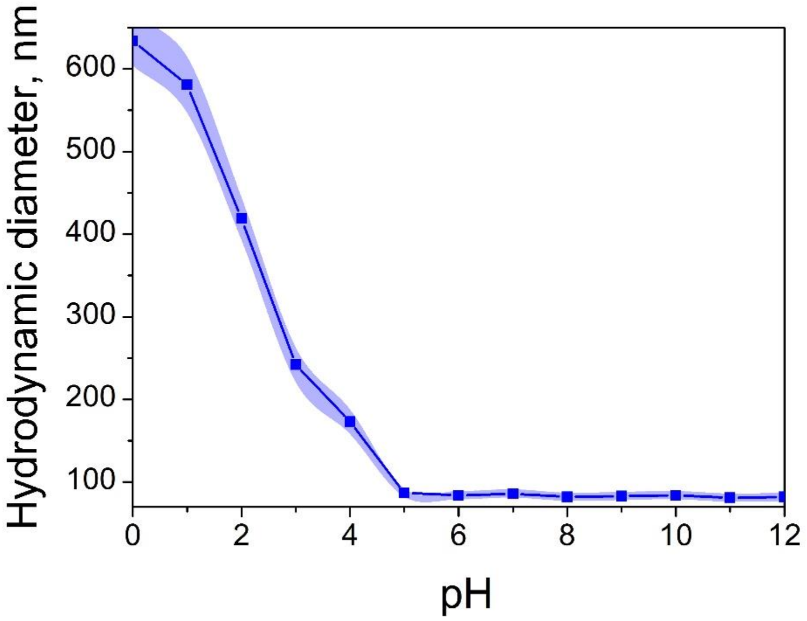

Figure 4S. Hydrodynamic diameter as a function of pH.
